# Epitranscriptomic RNA Methylation in Plant Development and Abiotic Stress Responses

**DOI:** 10.3389/fpls.2019.00500

**Published:** 2019-04-17

**Authors:** Jianzhong Hu, Stefano Manduzio, Hunseung Kang

**Affiliations:** Department of Applied Biology, College of Agriculture and Life Sciences, Chonnam National University, Gwangju, South Korea

**Keywords:** abiotic stress, epitranscriptome, RNA metabolism, RNA methylation, RNA modification

## Abstract

Recent advances in methylated RNA immunoprecipitation followed by sequencing and mass spectrometry have revealed widespread chemical modifications on mRNAs. Methylation of RNA bases such as *N*^6^-methyladenosine (m^6^A) and 5-methylcytidine (m^5^C) is the most prevalent mRNA modifications found in eukaryotes. In recent years, cellular factors introducing, interpreting, and deleting specific methylation marks on mRNAs, designated as “writers (methyltransferase),” “readers (RNA-binding protein),” and “erasers (demethylase),” respectively, have been identified in plants and animals. An emerging body of evidence shows that methylation on mRNAs affects diverse aspects of RNA metabolism, including stability, splicing, nucleus-to-cytoplasm export, alternative polyadenylation, and translation. Although our understanding for roles of writers, readers, and erasers in plants is far behind that for their animal counterparts, accumulating reports clearly demonstrate that these factors are essential for plant growth and abiotic stress responses. This review emphasizes the crucial roles of epitranscriptomic modifications of RNAs in new layer of gene expression regulation during the growth and response of plants to abiotic stresses.

## Introduction

Epigenetic regulation of gene expression via DNA methylation and histone modifications is an important strategy for living organisms to achieve fine-tuned regulation of developmental processes or responses to environmental cues. Similar to DNA methylation in epigenetic regulation, posttranscriptional RNA modifications are emerging as important “epitranscriptomic” regulatory networks in recent years (Saletore et al., 2012; Meyer and Jaffrey, 2014). Over 150 different chemical modifications on mRNAs, tRNAs, and rRNAs are currently known for all kingdoms of life (Cantara et al., 2010; Boccaletto et al., 2018). Among diverse modifications found on mRNAs, N6-methyladenosine (m^6^A) is the most prevalent modification in both plants and animals (Liu and Pan, 2016; Covelo-Molares et al., 2018). Recent advances in methylation RNA sequencing (Met RNA-seq) and deep RNA sequencing have revealed transcriptome-wide m^6^A methylation patterns in plants as well as in animals (Luo et al., 2014; Wang et al., 2015a; Cui et al., 2017). These modifications within mRNAs can affect multiple steps of transcript’s fate, including splicing (Haussmann et al., 2016; Xiao et al., 2016), nucleus-to-cytoplasm export (Zheng et al., 2013), RNA turnover (Du et al., 2016; Mauer et al., 2017; Wei et al., 2018), and translation (Meyer et al., 2015; Wang et al., 2015b; Choi et al., 2016).

**FIGURE 1:**
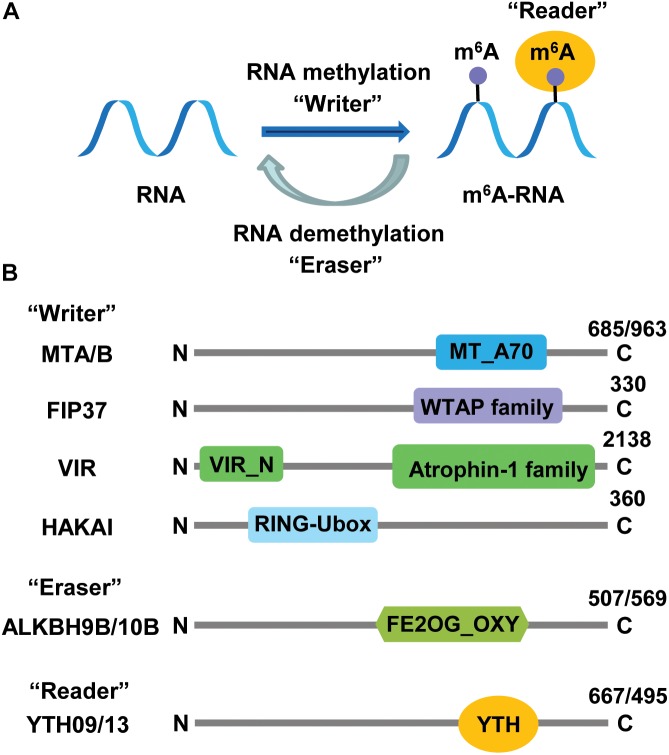
Roles and structural characteristics of m^6^A RNA methylation-related proteins. **(A)** Cellular factors introducing, deleting, and interpreting m^6^A marks are methyltransferase (“writers”), demethylase (“eraser”), and RNA-binding protein (“reader”), respectively. **(B)** The writer complex consists of five components: MTA/B (methyltransferase A/B), FIP37 (FKBP12 interacting protein 37), VIR (Virilizer), HAKAI (for “destruction” in Japanese, a c-Cb1-like protein), erasers belong to AlkB-homology (ALKBH) family proteins, and readers are YT512-B homology domain (YTHD) proteins. Numbers at the C terminus indicate the number of amino acid residues in each *Arabidopsis* protein. MT_A70, methytransferase_A70; FE2OG_OXY, Fe^2+^ 2-oxoglutarate dioxygenase domain; WTAP, WT1-associated protein.

The level and status of RNA methylation in cells depend on two crucial proteins: RNA methyltransferase (MT) designated as “writer” and RNA demethylase (DMT) designated as “eraser” (Figure 1). In addition to these two essential proteins required for the addition and removal of methyl groups on RNAs, a third protein designated as “reader” is involved in the recognition and processing of methylated RNAs (reviewed in Meyer and Jaffrey, 2014). In animals, genes encoding m^6^A writer (Ping et al., 2014; Schwartz et al., 2014), reader (Luo and Tong, 2014; Xu et al., 2014), and eraser (Jia et al., 2011; Zheng et al., 2013) proteins have been identified and characterized (Table 1). Notably, mutants lacking specific m^6^A writer, reader, or eraser have displayed abnormal development and altered response to hypoxia and high temperatures, suggesting crucial roles of RNA methylation in animal development and adaptation to changing environmental cues (reviewed in Meyer and Jaffrey, 2014; Yue et al., 2015; Hsu et al., 2017).

**Table 1:** List of writers, readers, and erasers involved in RNA methylation in *Arabidopsis thaliana* and rice (*Oryza sativa*).

Type	Gene name	Arabidopsis gene ID	Target RNA	Function	Rice ortholog	Animal counterpart
Writers	MTA	At4g10760	m^6^A	Embryo development	LOC_Os02g45110	METTL3
	MTB	At4g09980	m^6^A	Embryo development	LOC_Os01g16180	METTL14
					LOC_Os03g05420	
					LOC_Os10g31030	
	FIP37	At3g54170	m^6^A	Development	LOC_Os06g27970	WTAP
	VIRILIZER	At3g05680	m^6^A	Development	LOC_Os03g35340	VIRMA
	HAKAI	At5g01160	m^6^A	Development	LOC_Os10g35190	
	TRM4A	At4g40000	m^5^C		LOC_Os08g37780	
	TRM4B	At2g22400	m^5^C	Root development	LOC_Os09g29630	
Readers	YTH01 (ECT11)	At1g09810	m^6^A m^1^A		LOC_Os01g22630	YTHDF1
	YTH02 (ECT9)	At1g27960			LOC_Os08g12760	YTHDF2
	YTH03(CPSF30)	At1g30460			LOC_Os06g46400	YTHDF3
	YTH04 (ECT7)	At1g48110			LOC_Os03g20180	YTHDC1
	YTH05 (ECT4)	At1g55500		Development	LOC_Os03g53670	YTHDC2
	YTH06 (ECT8)	At1g79270			LOC_Os01g48790	
	YTH07 (ECT1)	At3g03950			LOC_Os04g51940	
	YTH08 (ECT5)	At3g13060			LOC_Os08g44200	
	YTH09 (ECT2)	At3g13460		Trichome branching	LOC_Os07g07490	
	YTH10 (ECT6)	At3g17330			LOC_Os04g51950	
	YTH11	At4g11970				
	YTH12 (ECT10)	At5g58190			LOC_Os05g04000	
	YTH13 (ECT3)	At5g61020		Trichome branching	LOC_Os05g01520	
Erasers	ALKBH1A	At1g11780			LOC_Os03g60190	ALKBH1
	ALKBH1B	At3g14140			LOC_Os11g29690	
	ALKBH1C	At3g14160				
	ALKBH1D	At5g01780				
	ALKBH2	At2g22260			LOC_Os06g17830	ALKBH2
	ALKBH6	At4g20350			LOC_Os10g28410	ALKBH6
	ALKBH8	At1g36310	tRNA mcm^5^U		LOC_Os04g51360	ALKBH8
					LOC_Os11g43610	
	ALKBH8A	At1g31600	tRNA mcm^5^U			
	ALKBH8B	At4g02485				
	ALKBH9A	At1g48980			LOC_Os06g04660	ALKBH5
	ALKBH9B	At2g17970	m^6^A	Viral infection		
	ALKBH9C	At4g36090				
	ALKBH10A	At2g48080			LOC_Os05g33310	
	ALKBH10B	At4g02940	m^6^A	Flowering	LOC_Os10g02760	


Although these recent studies clearly point to the importance of RNA methylation and essential roles of writers, readers, and erasers in the development of animals, functions of these proteins in plants are just beginning to be uncovered. *Arabidopsis* contains functional orthologs of m^6^A writer complex components, erasers, and reader proteins, some of which have been found to play essential roles in normal plant development (Bodi et al., 2012; Shen et al., 2016; Růžička et al., 2017; Arribas-Hernández et al., 2018; Scutenaire et al., 2018; Wei et al., 2018). All these aforementioned studies have emphasized the essential roles of RNA methylation in plant development. However, the identity and functions of most writers, readers, and erasers in plants are currently unclear. In this review, we systematically identified potential m^6^A writers, readers, and erasers in *Arabidopsis* and rice (*Oryza sativa*) by comparing sequence homology to animal counterparts. We also reviewed multiple functions and potential significance of m^6^A RNA methylation in the development and response of plants to diverse abiotic stresses.

## Diverse Modifications for Eukaryotic RNAs

Over 150 different internal modifications on RNAs have been identified (Cantara et al., 2010; Boccaletto et al., 2018), with different degree, topology, and kinds of modifications between mRNAs, tRNAs, and rRNAs. For instance, approximately 17% of total nucleotides in tRNAs are modified, whereas only 2% of nucleotides in rRNAs are modified (Jackman and Alfonzo, 2013). Among diverse modifications identified for tRNAs and rRNAs, 2^′^-O-ribose methylation and pseudouridilation of rRNAs and 5-methylcytosine (m^5^C) and 1-methylguanidine (m^1^G) of tRNAs are the most abundant (Chou et al., 2017). Despite emerging roles of mRNA modifications in its processing and function, mRNA is less densely modified compared to tRNAs and rRNAs (Gilbert et al., 2016). Only a handful of different methylations have been identified so far in mRNAs, with N6-methyladenosine (m^6^A) being the most abundant (Liu and Pan, 2016; Covelo-Molares et al., 2018). These methylations of bases can influence the structure of RNAs by increasing its hydrophobicity and disrupting the canonical Watson-Crick base pairing (Oerum et al., 2017; Väre et al., 2017).

Importantly, all organisms have evolved to cluster methylation marks in functionally critical positions rather than randomly distributing them along RNA molecules. Most of these modified bases in rRNAs are located at the interface between ribosomal large and small subunits corresponding to P-site and A-site (Sharma and Lafontaine, 2015; Sloan et al., 2017). Wobble positions 34 and 37 of the anticodon loop in tRNAs are the most frequently and diversely modified (Väre et al., 2017). These conserved modification patterns reflect the essential role of RNA methylation in ribosome structure and biogenesis, codon recognition and decoding, and translation initiation or elongation (Jackman and Alfonzo, 2013; Chou et al., 2017; Sloan et al., 2017; Väre et al., 2017). Similar to rRNAs and tRNAs, mRNAs are also methylated in specific regions. For instance, m^6^A maps preferentially to the transcription start site, the stop codon, and the 3^′^ UTR (Dominissini et al., 2012; Luo et al., 2014; Meyer and Jaffrey, 2014), while m^5^C is predominantly found in 3^′^ UTR and coding regions (Squires et al., 2012; David et al., 2017). Several studies have shown that m^1^A methylation is frequently found in the start codon and the first splicing site which influences translation (Dominissini et al., 2016; Safra et al., 2017). Clearly, the degree, topology, and non-random distribution of RNA modifications are crucial for its specific cellular functions.

## Writers, Erasers, and Readers Involved in m^6^A RNA Methylation and Recognition

### Writers

Genes encoding m^6^A writer complexes have been identified and characterized firstly in animals. Several proteins including methyltransferase-like 3 (METTL3) and METTL14, Wilms’ tumor 1-associating protein (WTAP), and Vir like m^6^A methyltransferase-associated (VIRMA; KIAA1429) are known to form multicomponent m^6^A writer complexes in animals (Shah and Clancy, 1992; Ping et al., 2014; Schwartz et al., 2014; Table 1).

Methyltransferase-like 3 is the principal enzyme exerting methyltransferase activity, while METTL14 has a supporting role forming a METTL3-METTL14 heterodimer (Sledz and Jinek, 2016; Wang et al., 2016). After the identification of METTL3 in mammals as a homolog of yeast methyltransferase IME4 (Shah and Clancy, 1992), its orthologs were identified in different species including *Arabidopsis* and Drosophila. At present, *Arabidopsis* orthologs of animal m^6^A writer components have been identified, including MTA (ortholog of METTL3) and MTB (ortholog of METTL14).

Wilms’ tumor 1-associating protein functions as a stabilizer for the heterodimer localized in nuclear speckle (Ping et al., 2014; Lence et al., 2016). VIRMA plays a role in guiding the methyltransferase complex to the selective target region of mRNAs (Niessen et al., 2001; Yue et al., 2018). *Arabidopsis* VIR and FIP37 were identified as a ortholog of VIRMA and WTAP, respectively (Zhong et al., 2008; Bodi et al., 2012; Shen et al., 2016; Růžička et al., 2017).

Recently, zinc finger CCCH domain-containing protein 13 (ZC3H13), the latest component of methyltransferase complex, was found to be essential for localization of methyltransferase complex in mammals and *Drosophila* (Guo et al., 2018; Knuckles et al., 2018). However, the existence and molecular function of ZC3H13 in plants remain unknown. Interestingly, *Arabidopsis* contains E3 ubiquitin ligase HAKAI as an additional m^6^A writer component (Růžička et al., 2017; Table 1). Although knockdown of its expression can decrease m^6^A level (Růžička et al., 2017), the primary role of HAKAI in methyltransferase complexes has yet to be investigated.

### Erasers

Removal of methylation marks on RNAs is carried out by α-ketoglutarate-dependent dioxygenase (AlkB) homolog (ALKBH) proteins that can erase alkyl and methyl groups from DNAs, RNAs, and proteins (Fedeles et al., 2015; Alemu et al., 2016). Mammals have nine ALKBH family members: ALKBH1 to ALKBH8 and fat mass- and obesity-associated protein (FTO) (Ougland et al., 2015; Table 1). Although ALKBH2 and ALKBH3 have been identified as main DNA repair enzymes, ALKBH3 also shows activity on m^1^A and m^3^C of RNAs (Ueda et al., 2017). Interestingly, ALKBH1 acts on a wide range of substrates in DNAs, RNAs, and histones (Westbye et al., 2008; Ougland et al., 2012; Wu et al., 2016). In addition to its role in cytoplasm, human ALKBH1 targets several m^1^A methylated tRNAs in mitochondria, influencing the organellar translation and function (Kawarada et al., 2017; Müller et al., 2018). ALKBH8, another tRNA DMT, interestingly contains both methyltransferase and demethylase domains, unlike other family members (Pastore et al., 2012).

Only two m^6^A erasers, ALKBH5 and FTO, have been identified in animals so far. Both enzymes were originally shown to be involved in demethylation of m^6^A (Jia et al., 2011; Zheng et al., 2013). However, recent studies have suggested that FTO has a much higher activity toward N^6^, 2^′^-O-dimethyladenosine (m^6^A_m_) compared to that for m^6^A (Meyer and Jaffrey, 2017; Mauer et al., 2017; Mauer and Jaffrey, 2018). ALKBH5 and FTO have been found to be involved in alternative splicing, 3^′^-UTR processing, mRNA stability, translation, and amino-acids deprivation response pathway (Zheng et al., 2013; Zhao et al., 2014; Bartosovic et al., 2017; Tang et al., 2018). *Arabidopsis* contains several putative m^6^A eraser ALKBH family proteins (Table 1), among which only two eraser proteins ALKBH9B and ALKBH10B have been functionally characterized in viral infection and floral transition (Duan et al., 2017; Martínez-Pérez et al., 2017). In summary, although increasing number of erasers targeting specific methylation marks have been identified, the activity and substrate RNAs of most ALKBH family members in plants and animals are yet to be determined.

### Readers

Interpretation of methylation marks is tightly related to posttranscriptional regulation of mRNA metabolism which requires reader proteins to recognize methylated transcripts and ultimately determine their fates. In recent years, several RNA-binding proteins (RBPs) that can recognize m^6^A marks on mRNAs have been identified in animals by RNA-protein immunoprecipitation using synthetic m^6^A-containing RNAs (Dominissini et al., 2012; Xu et al., 2014; Arguello et al., 2017; Edupuganti et al., 2017; Wu et al., 2017). YT521-B homology (YTH) domain family (YTHDF) protein was first identified as an m^6^A-binding protein (Xu et al., 2014). Recently, human and mouse YTHDF proteins including YTHDF1, YTHDF2, YTHDF3, YTHDC1, and YTHDC2 were found to possess a specific binding pocket for m^6^A nucleotides and exhibit significantly high affinity to methylated RNAs, suggesting their role as m^6^A readers (Dominissini et al., 2012; Hsu et al., 2017; Xiang et al., 2017; Liao et al., 2018). YHHDF2 can bind to m^6^A-modified RNAs and play a distinct role in mRNA degradation by recruiting the CCR4-NOT deadenylase complex (Wang et al., 2014; Zhou et al., 2015; Du et al., 2016). YTHDF1 was found to recognize the 5^′^UTR of m^6^A-modified mRNAs in the cytosol, which promotes translation of target transcripts in a cap-independent manner (Wang et al., 2015b; Shi et al., 2017). YTHDC1 is involved in exon-selective gene splicing in the nucleus (Xiang et al., 2017). Interestingly, YTHDC2 also contains RNA helicase domain (Jain et al., 2018). *Arabidopsis* and rice genomes encode 13 and 12 YTHD proteins, respectively (Li et al., 2014a; Table 1). Contrary to extensive study on YTHD proteins in animals, only three evolutionarily conserved c-terminal region (ECT) family proteins have recently been functionally characterized in *Arabidopsis* as YTHD homologs (Arribas-Hernández et al., 2018; Scutenaire et al., 2018; Wei et al., 2018; Table 1).

Besides YTHD proteins, two other proteins containing different RNA-binding domains that can recognize m^6^A marks in animal cells have been reported. One is a heterogeneous nuclear ribonucleoprotein A2/B1 (HNRNPA2B1) which regulate RNA splicing in the nucleus through a well-characterized RNA-recognition motif (Alarcon et al., 2015). Notably, instead of directly binding to m^6^A site as YTHD proteins, HNRNPA2B1 might bind to altered structures right after the m^6^A site (Alarcon et al., 2015). Insulin-like growth factor 2 mRNA-binding protein (IGF2BP) contains tandem K-homology (KH) domains to recognize m^6^A sites and enhance target mRNA stability, storage, and translation in an m^6^A-dependent manner (Nicastro et al., 2015; Huang et al., 2018). Eukaryotic initiation factor 3 (eIF3) can also promote translation of mRNAs depending on m^6^A modification (Meyer et al., 2015). Clearly, more reader proteins recognizing other RNA modifications as well as m^6^A marks should be uncovered to fully understand cellular roles of epitranscriptomic RNA modifications in both plants and animals.

## RNA Methylation in Animal Development and Diseases

**FIGURE 2:**
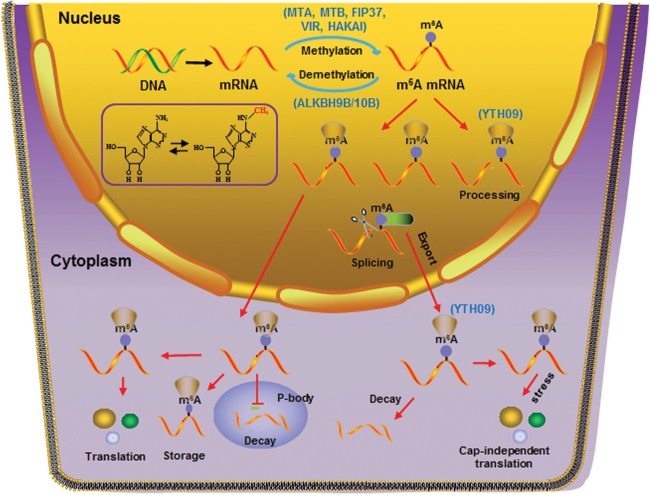
Diverse cellular processes affected by m^6^A RNA methylation. Splicing of mRNAs in the nucleus and diverse RNA metabolism in cytoplasm, including cap-dependent and cap-independent translation, RNA decay in cytosol and P-body, and RNA storage, is affected by m^6^A RNA methylation. Specific “reader” proteins recognizing m^6^A marks on mRNAs play essential roles in these cellular processes. Writers (MTA, MTB, FIP37, VIR, and HAKAI), erasers (ALKBH9B/10B), and reader (YTH09) identified in *Arabidopsis* are shown.

m^6^A methylation has been demonstrated to affect all fates of mRNA metabolism, including pre-mRNA processing and intron splicing in the nucleus, nucleus-to cytoplasm RNA export, translation, and RNA decay in the cytoplasm (Figure 2). Analysis of different *mettl* mutants demonstrated the essential role of m^6^A methylation in cell development, proliferation, differentiation, and motility by regulating mRNA stability and splicing pattern of diverse transcripts (Wang et al., 2014; Chen et al., 2015; Geula et al., 2015; Park and Hong, 2017; Widagdo and Anggono, 2018). Loss of FTO can inhibit differentiation of primary myoblasts and skeletal muscle in mice, suggesting that m^6^A demethylase FTO plays a crucial role in somatic and neural stem cell differentiation (Wang et al., 2017). A larger number of gene encoding clock genes and clock output genes are enriched in m^6^A methylation (Fustin et al., 2013; Hastings, 2013) and changes in m^6^A levels can affect circadian rhythms, cellular growth, and survival (Fustin et al., 2018).

Notably, recent studies have demonstrated that alteration in m^6^A levels is closely associated with various diseases, especially cancer (reviewed in Dai et al., 2018; Pan et al., 2018). For example, FTO affects m^6^A level and translation of *Angptl4* mRNA, which regulates fatty acid mobilization in adipocytes and body weight (Wang et al., 2015a). Low m^6^A level in total RNA is related to type 2 diabetes mellitus (Shen et al., 2014). Considering that aberrant cell growth and differentiation cause cancer, it is worth noting that cancer cells may improve their survival rate and progression by modulating aberrant methylation of target RNAs. Several studies have shown that expression of FTO or ALKBH5 can decrease m^6^A level, resulting in enhanced cancer cell growth (Zhang et al., 2016; Li et al., 2017; Zhang et al., 2017). METTL3 acts as an oncogene in cancer cells, enhancing the translation of cancer-inducing genes by interacting with translation initiation factor (Lin et al., 2016).

In addition to m^6^A methylation, m^5^C is also involved in cell development and diseases. This modification is deeply associated with testis differentiation and tumor cell proliferation. A previous study has shown that NOP2/sun RNA methyltransferase family member 2 (NSUN2), an m^5^C writer, is highly expressed in tumor cells and its depletion decreases levels of Ddx4, Miwi, and Tudor domain-containing proteins, suggesting an essential role of m^5^C RNA methylation in male germ cell differentiation (Frye and Watt, 2006). Moreover, loss of NSUN2 causes an accumulation of progenitors, decreases in upper-layer neurons, and increases in tRNA fragment accumulation in the brain, resulting in damage to neural stem cell differentiation and motility (Flores et al., 2017). Although these studies clearly demonstrate the importance of m^6^A and m^5^C in cell proliferation and diseases, biological functions of other RNA methylations in animal development and pathogenesis are yet to be elucidated.

## RNA Methylation in Plant Development and Abiotic Stress Responses

Although our understanding of writers, readers, and erasers in plants is far behind their animal counterparts, accumulating reports clearly demonstrate that these factors are essential for plant growth and abiotic stress responses. Herein, we will summarize and discuss characterized and potential writers, readers, and erasers (Table 1) in plants.

### m^6^A Writers

Genome-wide m^6^A methylation patterns have been mapped in barley, *Arabidopsis*, and rice (Li et al., 2014b; Luo et al., 2014). However, key enzymes responsible for this methylation have only been studied in Arabidopsis. Analysis of *mta* (*Arabidopsis* ortholog of human METTL3) knockdown mutants has revealed that MTA is required for m^6^A mRNA methylation and essential for normal growth and development, such as shoot and root growth as well as leaf and floral development (Zhong et al., 2008; Bodi et al., 2012). Moreover, MTA was found to interact with MTB, an *Arabidopsis* ortholog of human METTLl4. Knockdown of MTB showed a similar but less severe phenotype compared to *mta* mutants, indicating that both writers are essential for plant development (Růžička et al., 2017). The *Arabidopsis* m^6^A writer complex also includes an ortholog of human WTAP named FIP37. Depletion of FIP37 results in embryo lethality while its partial loss causes huge overproliferation of shoot meristems by increasing the stability of *shootmeristemless* (*STM*) and *WUSCHEL (WUS)* (Shen et al., 2016). Vir and Hakai are other m^6^A writer components in *Arabidopsis*. They affects root and shoot growth as well as cotyledon development, similar to other m^6^A writer mutant phenotypes (Růžička et al., 2017). However, the molecular mechanism underlying Vir and Hakai functions is yet to be elucidated.

**FIGURE 3:**
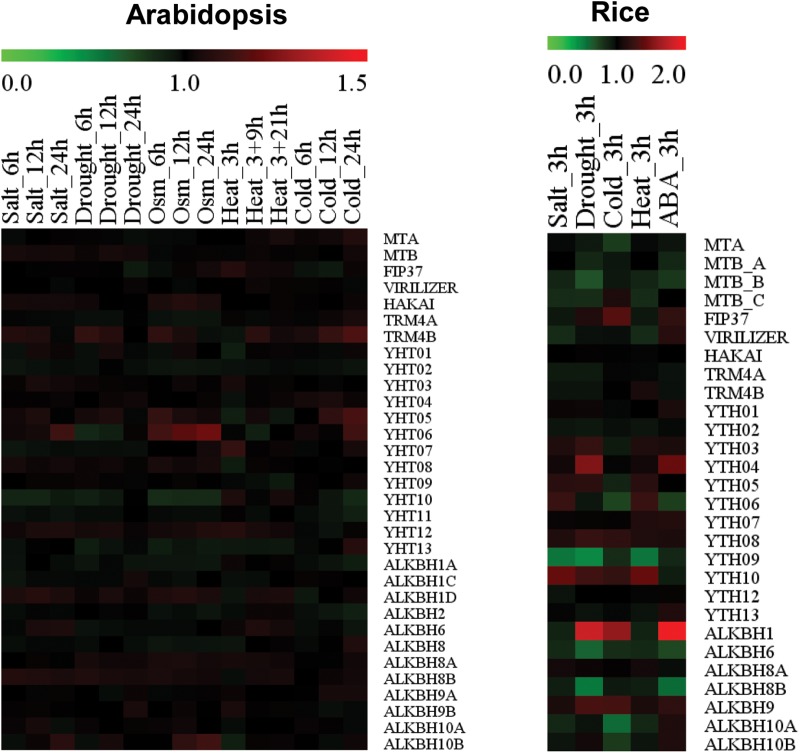
Heatmap showing stress-responsive expression patterns of writers, readers, and erasers in Arabidopsis and rice. Red or green colors represent upregulated and downregulated expression level, respectively. Microarray data were obtained from GENEVESTIGATOR, and expression levels of each gene under stress conditions were calculated relative to control levels.

Despite increasing understanding of the roles of m^6^A writers in plant growth and development, reports demonstrating their involvement and functions in plant response to abiotic stresses are lacking. Our analysis of publically available microarray data using GENEVESTIGATOR revealed that expressions levels of writers in *Arabidopsis* and rice are differently modulated by diverse abiotic stresses (Figure 3). In *Arabidopsis*, levels of most m^6^A writer components were not significantly modulated by abiotic stresses. Levels of *MTA* and *FIP37* were only marginally increased by cold and heat stress, respectively. In rice, the level of *OsFIP* was increased by cold stress whereas levels of *OsMTA*, *OsMTB*, and *OsVIR* were decrease by cold, drought, or salt stress. The constant expression of m^6^A writer components under normal and stress conditions suggests the fundamental role of m^6^A methylation in plant development and stress responses.

### m^5^C Writers

Although m^5^C methylation in DNA has been studied for many years, its cellular and molecular functions in RNAs is just beginning to be uncovered. Due to advancement in RNA sequencing, m^5^C RNA methylation could be mapped to mRNAs in both animals and plants (Schaefer et al., 2009; Hussain et al., 2013; Song et al., 2018). Overall, m^5^C RNA methylation is a less abundant modification of mRNA than m^6^A methylation. In *Arabidopsis*, two enzymes, TRM4A and TRM4B, are responsible for m^5^C RNA methylation. Both enzymes are orthologs of human m^5^C methyltransferase NSuns2. However, TRM4A contributes to tRNA m^5^C methylation while TRM4B targets mRNA for m^5^C methylation. Loss of TRM4A does not exhibit any visible phenotype while loss of Trm4B reduces root length, suggesting the role of mRNA m^5^C methylation in root development regulation (David et al., 2017). In accordance to this, loss of m^5^C RNA methylation affects the stability of short hypocotyl 2 (SHY2) and indoleacetic acid-induced protein 16 (IAA16), two critical genes related to root development (Cui et al., 2017). Our analysis showed that expression levels of *Arabidopsis* *TRM4B* are marginally increased by cold stress, although they decrease under heat stress. In contrast, expression levels of rice *TRM4A* and *TRM4B* are not altered in response to abiotic stresses (Figure 3). Although these expression patterns suggest potential roles of m^5^C writers in abiotic stress response, the relevance of m^5^C methylation to abiotic stress responses awaits further investigation.

### m^6^A Erasers

Among protein factors involved in RNA methylation in plants, erasers are so far the least studied, although new knowledge is gained rapidly. Thirteen *Arabidopsis* ALKBH family members have been identified by bioinformatic analysis (Mielecki et al., 2012). However, only a few of them have been studied so far (Table 1). Among them, ALKBH9A, 9B, 9C, 10A, and 10B show the highest amino acid sequence similarity with human ALKBH5. Other family members are numbered based on their sequence similarity to human orthologs (Table 1). Like animal counterparts, most erasers are localized in the nucleus and cytoplasm whereas ALKBH1D is also present in chloroplasts. Interestingly, some of them show relocalization to the nucleus in response to methylating agents (Mielecki et al., 2012). ALKBH10B was identified as the principal mRNA m^6^A eraser influencing floral transition by controlling transcript levels of *SPL3*, *SPL9*, and *FLOWERING LOCUS T* (Duan et al., 2017). Another demethylase, ALKBH9B, was shown to revert m^6^A from single-stranded RNA *in vitro* (Martínez-Pérez et al., 2017). Although *alkbh9b* knockout mutants do not show differences in plant RNA m^6^A methylation level (Duan et al., 2017), its depletion results in hypermethylation of alfalfa mosaic virus (AMV) RNA, mediating systemic infection by interacting with viral cap proteins (Martínez-Pérez et al., 2017).

Expression of *ALKBH9A* is highly induced in roots under salt stress but not in response to ABA (Ma et al., 2006). Its level is much lower than ALKBH9 and ALKBH10 under normal conditions (Duan et al., 2017). ALKBH10A is down-regulated by heat stress (Merret et al., 2015) whereas ALKBH10B is up-regulated in response to karrikins (Nelson et al., 2010). Although these previous studies suggest a specific role of ALKBHs in stress responses as well as plant development, nothing is known about their actual roles. Our analysis showed that expression levels of *ALKBH* members were marginally up- or down-regulated in *Arabidopsis* by different abiotic stresses (Figure 3). Notably, levels of *ALKBH1* in rice were highly increased upon drought, cold, or ABA treatment whereas expression levels of *ALKBH6*, *ALKBH8B*, and *ALKBH10A* were decreased by drought, ABA, or cold (Figure 3). These data suggest that ALKBHs could play important roles in abiotic stress responses, although this awaits further investigation.

### m^6^A Readers

Although several RBPs interpreting m^6^A marks have been identified in animals, roles of only three YTHD m^6^A reader proteins have very recently been determined in *Arabidopsis* (Arribas-Hernández et al., 2018; Scutenaire et al., 2018; Wei et al., 2018). YTHD09 (ECT2) is involved in trichome development. Moreover, cytoplasmic-localized YTHD09 relocates to stress granules upon heat exposure, suggesting its role in mRNA fate control under stress conditions (Scutenaire et al., 2018). By using single and double mutants, it has been demonstrated that YTHD09 (ECT2), YTHD13 (ECT3), and ECT4 regulate the timing and execution of plant organogenesis (Arribas-Hernández et al., 2018). Moreover, a molecular study revealed that ECT2 targets a large number of m^6^A-containing transcripts, including *TTG1*, *ITB1*, and *DIS2*, which are involved in trichome development (Wei et al., 2018). Further sequencing analysis suggested that ECT2 increases the stability of these transcripts and influences trichome development (Wei et al., 2018). Although these studies clearly point to important roles of YTHD readers in plant development, more in-depth and focused efforts are needed to identify and characterize potential reader proteins (Table 1) that can recognize not only m^6^A modification, but also other methylation marks in plants.

No reports demonstrating the involvement or functions of any RNA methylation reader proteins in plant response to abiotic stresses have been published so far. However, a previous study and our current analysis showed that the expression of *YTHDs* in *Arabidopsis* and rice is highly regulated by different abiotic stresses (Li et al., 2014a; Figure 3). In *Arabidopsis*, levels of *YTHD05*, *YTHD06*, and *YTHD07* are increased by heat, cold, hypoxia, or submergence stress. In contrast, the expression level of *YTHD10* decreases under cold, drought, salt, or osmotic stress whereas *YTHD08* level is reduced by heat stress. In rice, *YTHDs* responded differently to various abiotic stresses (Li et al., 2014a; Figure 3). Expression levels of *YTHD05, YTHD06*, *YTHD07*, and *YTHD09* are downregulated by cold stress whereas levels of *YTHD03* and *YTHD08* increase under submergence and heat stress, respectively. Notably, none of these rice *YTHDs* showed altered expression under salt stress whereas *YTHD01*, *YTHD02*, *YTHD03*, *YTHD04*, or *YTHD08* does not respond to cold stress. The fact that m^6^A reader proteins respond more to abiotic stresses than writers and erasers suggests that decoding of methylation marks is much more important than introducing or removing these marks during stress adaptation process in plants. It would be interesting to characterize roles of reader proteins in RNA metabolism and its consequence in stress responses.

## Concluding Remarks and Perspectives

Chemical modifications of RNAs are invaluable ways to expand decoding capacity of RNA transcripts beyond genetic information inherent to genome sequences. They are crucial for posttranscriptional gene regulatory events such as mRNA splicing, stability, and translation. The ability to regulate the fate of RNA molecules through nucleotide modifications is vital to plant survival and fitness under adverse as well as favorable environmental conditions. Despite the increasing discovery of cellular components essential for chemical modification and decoding of modified RNA molecules, our knowledge regarding physiological roles of proteins involved in these processes is far from sufficient. Several key questions remain to be further investigated. Are there any other internal RNA modifications not identified so far? How these components are regulated depending on developmental stages and/or in response to changing environmental cues? What guides the specificity of interactions between these components with target transcripts? Are these components conserved between dicots and monocots, especially in crop species? Addressing these questions will greatly expand our knowledge on the process of chemical modifications of RNAs and its effects on plant survival and fitness under stressful conditions. Such studies could provide potential new targets for engineering crop plants with higher adaptability to adverse environmental conditions.

## Author Contributions

HK designed the concept. JH and SM compiled and analyzed data. JH, SM, and HK contributed to the writing of this review.

## Conflict of Interest Statement

The authors declare that the research was conducted in the absence of any commercial or financial relationships that could be construed as a potential conflict of interest.

## References

[B1] Alarcon C. R. Goodarzi H. Lee H. Liu X. Tavazoie S. Tavazoie S. F. (2015). HNRNPA2B1 is a mediator of m^6^A-dependent nuclear RNA processing events. *Cell* 162 1299–1308. 10.1016/j.cell.2015.08.011 26321680 PMC4673968

[B2] Alemu E. A. He C. Klungland A. (2016). ALKBHs-facilitated RNA modifications and de-modifications. *DNA Repair* 44 87–91. 10.1016/j.dnarep.2016.05.026 27237585 PMC5120542

[B3] Arguello A. E. Deliberto A. N. Kleiner R. (2017). RNA chemical proteomics approach reveals the *N*^6^-methyladenosine (m^6^A)-regulated protein-RNA interactome. *J. Am. Chem. Soc.* 139 17249–17252. 10.1021/jacs.7b09213 29140688

[B4] Arribas-Hernández L. Bressendorff S. Hansen M. H. Poulsen C. Erdmann S. Brodersen P. (2018). An m^6^A-YTH module controls developmental timing and morphogenesis in Arabidopsis. *Plant Cell* 30 952–967. 10.1105/tpc.17.00833 29643069 PMC6002192

[B5] Bartosovic M. Molares H. C. Gregorova P. Hrossova D. Kudla G. Vanacova S. (2017). *N*^6^-methyladenosine demethylase FTO targets pre-mRNAs and regulates alternative splicing and 3′-end processing. *Nucleic Acids Res.* 45 11356–11370. 10.1093/nar/gkx778 28977517 PMC5737695

[B6] Boccaletto P. Machnicka M. A. Purta E. Piatkowski P. Baginski B. Wirecki T. K. (2018). MODOMICS: a database of RNA modification pathways. 2017 update. *Nucleic Acids Res.* 46 D303–D307. 10.1093/nar/gkx1030 29106616 PMC5753262

[B7] Bodi Z. Zhong S. Mehra S. Song J. Graham N. Li H. (2012). Adenosine methylation in *Arabidopsis* mRNA is associated with the 3′ end and reduced levels cause developmental defects. *Front. Plant Sci.* 3:48. 10.3389/fpls.2012.00048 22639649 PMC3355605

[B8] Cantara W. A. Crain P. F. Rozenski J. McCloskey J. A. Harris K. A. Zhang X. (2010). The RNA modification database RNAMDB: 2011 update. *Nucleic Acids Res.* 39 D195–D201. 10.1093/nar/gkq1028 21071406 PMC3013656

[B9] Chen T. Hao Y. J. Zhang Y. Li M. M. Wang M. Han W. (2015). m^6^A RNA methylation is regulated by microRNAs and promotes reprogramming to pluripotency. *Cell Stem Cell* 16 289–301. 10.1016/j.stem.2015.01.016 25683224

[B10] Choi J. Ieong K. W. Demirci H. Chen J. Petrov A. Prabhakar A. (2016). *N*^6^-methyladenosine in mRNA disrupts tRNA selection and translation-elongation dynamics. *Nat. Struct. Mol. Biol.* 23 110–115. 10.1038/nsmb.3148 26751643 PMC4826618

[B11] Chou H. J. Donnard E. Gustafsson H. T. Garber M. Rando O. J. (2017). Transcriptome-wide analysis of roles for tRNA modifications in translational regulation. *Mol. Cell* 68 978–992. 10.1016/j.molcel.2017.11.002 29198561 PMC5728682

[B12] Covelo-Molares H. Bartosovic M. Vanacova S. (2018). RNA methylation in nuclear pre-mRNA processing. *Wiley Interdiscip. Rev. RNA* 9:e1489. 10.1002/wrna.1489 29921017 PMC6221173

[B13] Cui X. Liang Z. Shen L. Zhang Q. Bao S. Geng Y. (2017). 5-Methylcytosine RNA methylation in *Arabidopsis thaliana*. *Mol. Plant* 10 1387–1399. 10.1016/j.molp.2017.09.013 28965832

[B14] Dai D. Wang H. Zhu L. Jin H. Wang X. (2018). *N*^6^-methyladenosine links RNA metabolism to cancer progression. *Cell Death Dis.* 9:124. 10.1038/s41419-017-0129-x 29374143 PMC5833385

[B15] David R. Burgess A. Parker B. Li J. Pulsford K. Sibbritt T. (2017). Transcriptome-wide mapping of RNA 5-methylcytosine in Arabidopsis mRNAs and noncoding RNAs. *Plant Cell* 29 445–460. 10.1105/tpc.16.00751 28062751 PMC5385953

[B16] Dominissini D. Moshitch-Moshkovitz S. Schwartz S. Salmon-Divon M. Ungar L. Osenberg S. (2012). Topology of the human and mouse m^6^A RNA methylomes revealed by m^6^A-seq. *Nature* 485 201–206. 10.1038/nature11112 22575960

[B17] Dominissini D. Nachtergaele S. Moshitch-Moshkovitz S. Peer E. Kol N. Benhaim M. S. (2016). The dynamic N1-methyladenosine methylome in eukaryotic messenger RNA. *Nature* 530 441–446. 10.1038/nature16998 26863196 PMC4842015

[B18] Du H. Zhao Y. He J. Zhang Y. Xi H. Liu M. (2016). YTHDF2 destabilizes m^6^A-containing RNA through direct recruitment of the CCR4–NOT deadenylase complex. *Nat. Commun.* 7:12626. 10.1038/ncomms12626 27558897 PMC5007331

[B19] Duan H.-C. Wei L.-H. Zhang C. Wang Y. Chen L. Lu Z. (2017). ALKBH10B is an RNA *N*^6^-methyladenosine demethylase affecting Arabidopsis floral transition. *Plant Cell* 29 2995–3011. 10.1105/tpc.16.00912 29180595 PMC5757257

[B20] Edupuganti R. R. Geiger S. Lindeboom R. Shi H. Hsu P. J. Lu Z. (2017). *N*^6^-methyladenosine (m^6^A) recruits and repels proteins to regulate mRNA homeostasis. *Nat. Struct. Mol. Biol.* 24 870–878. 10.1038/nsmb.3462 28869609 PMC5725193

[B21] Fedeles B. I. Singh V. Delaney J. C. Li D. Essigmann J. M. (2015). The AlkB family of Fe(II)/α-ketoglutarate-dependent dioxygenases: repairing nucleic acid alkylation damage and beyond. *J. Biol. Chem.* 290 20734–20742. 10.1074/jbc.R115.656462 26152727 PMC4543635

[B22] Flores J. V. Cordero-Espinoza L. Oeztuerk-Winder F. Andersson-Rolf A. Selmi T. Blanco S. (2017). Cytosine-5 RNA methylation regulates neural stem cell differentiation and motility. *Stem Cell Rep.* 8 112–124. 10.1016/j.stemcr.2016.11.014 28041877 PMC5233436

[B23] Frye M. Watt F. M. (2006). The RNA methyltransferase Misu (NSun2) mediates Myc-induced proliferation and is upregulated in tumors. *Curr. Biol.* 16 971–981. 10.1016/j.cub.2006.04.027 16713953

[B24] Fustin J. M. Doi M. Yamaguchi Y. Hida H. Nishimura S. Yoshida M. (2013). RNA-methylation-dependent RNA processing controls the speed of the circadian clock. *Cell* 155 793–806. 10.1016/j.cell.2013.10.026 24209618

[B25] Fustin J. M. Kojima R. Itoh K. Chang H. Y. Ye S. Zhuang B. (2018). Two Ck1δ transcripts regulated by m^6^A methylation code for two antagonistic kinases in the control of the circadian clock. *Proc. Natl. Acad. Sci. U.S.A.* 115 5980–5985. 10.1073/pnas.1809838115 29784786 PMC6003373

[B26] Geula S. Moshitch-Moshkovitz S. Dominissini D. Mansour A. A. Kol N. Salmon-Divon M. (2015). m^6^A mRNA methylation facilitates resolution of naive pluripotency toward differentiation. *Science* 347 1002–1006. 10.1126/science.1261417 25569111

[B27] Gilbert W. V. Bell T. A. Schaening C. (2016). Messenger RNA modifications: form, distribution and function. *Science* 352 1408–1412. 10.1126/science.aad8711 27313037 PMC5094196

[B28] Guo J. Tang H.-W. Li J. Perrimon N. Yan D. (2018). Xio is a component of the *Drosophila* Sex determination pathway and RNA *N*^6^-methyladenosine methyltransferase complex. *Proc. Natl. Acad. Sci. U.S.A.* 115 3674–3679. 10.1073/pnas.1720945115 29555755 PMC5889661

[B29] Hastings M. H. (2013). m^6^A mRNA methylation: a new circadian pacesetter. *Cell* 155 740–741. 10.1016/j.cell.2013.10.028 24209613

[B30] Haussmann I. U. Bodi Z. Sanchez-Moran E. Mongan N. P. Archer N. Fray R. G. (2016). m^6^A potentiates *Sxl* alternative pre-mRNA splicing for robust *Drosophila* sex determination. *Nature* 540 301–304. 10.1038/nature20577 27919081

[B31] Hsu P. J. Zhu Y. Ma H. Guo Y. Shi X. Liu Y. (2017). Ythdc2 is an *N*^6^-methyladenosine binding protein that regulates mammalian spermatogenesis. *Cell Res.* 27 1115–1127. 10.1038/cr.2017.99 28809393 PMC5587856

[B32] Huang H. Weng H. Sun W. Qin X. Shi H. Wu H. (2018). Recognition of RNA *N*^6^-methyladenosine by IGF2BP proteins enhances mRNA stability and translation. *Nat. Cell Biol.* 20 285–296. 10.1038/s41556-018-0045-z 29476152 PMC5826585

[B33] Hussain S. Tuorto F. Menon S. Blanco S. Cox C. Flores J. V. (2013). The mouse cytosine-5 RNA methyltransferase NSun2 is a component of the chromatoid body and required for testis differentiation. *Mol. Cell. Biol.* 33 1561–1570. 10.1128/MCB.01523-12 23401851 PMC3624257

[B34] Jackman J. E. Alfonzo J. D. (2013). Transfer RNA modifications: nature’s combinatorial chemistry playground. *Wiley Interdiscip. Rev. RNA* 4 35–48. 10.1002/wrna.1144 23139145 PMC3680101

[B35] Jain D. Puno M. R. Meydan C. Lailler N. Mason C. E. Lima C. D. (2018). *ketu* mutant mice uncover an essential meiotic function for the ancient RNA helicase YTHDC2. *eLife* 7:e30919. 10.7554/eLife.30919 29360036 PMC5832417

[B36] Jia G. Fu Y. Zhao X. Dai Q. Zheng G. Yang Y. (2011). *N*^6^-Methyladenosine in nuclear RNA is a major substrate of the obesity-associated FTO. *Nat. Chem. Biol.* 7 885–887. 10.1038/nchembio.687 22002720 PMC3218240

[B37] Kawarada L. Suzuki T. Ohira T. Hirata S. Miyauchi K. Suzuki T. (2017). ALKBH1 is an RNA dioxygenase responsible for cytoplasmic and mitochondrial tRNA modifications. *Nucleic Acids Res.* 45 7401–7415. 10.1093/nar/gkx354 28472312 PMC5499545

[B38] Knuckles P. Lence T. Haussmann I. U. Jacob D. Kreim N. Carl S. H. (2018). Zc3h13/Flacc is required for adenosine methylation by bridging the mRNA-binding factor Rbm15/Spenito to the m^6^A machinery component Wtap/Fl(2)d. *Genes Dev.* 32 415–429. 10.1101/gad.309146 29535189 PMC5900714

[B39] Lence T. Akhtar J. Bayer M. Schmid K. Spindler L. Ho C. H. (2016). m^6^A modulates neuronal functions and sex determination in *Drosophila*. *Nature* 540 242–247. 10.1038/nature20568 27919077

[B40] Li D. Zhang H. Hong Y. Huang L. Li X. Zhang Y. (2014a). Genome-wide identification, biochemical characterization, and expression analyses of the YTH domain-containing RNA-binding protein family in *Arabidopsis* and rice. *Plant Mol. Biol. Rep.* 32 1169–1186. 10.1007/s11105-014-0724-2

[B41] Li Y. Wang X. Li C. Hu S. Yu J. Song S. H. (2014b). Transcriptome-wide *N*^6^-methyladenosine profiling of rice callus and leaf reveals the presence of tissue-specific competitors involved in selective mRNA modification. *RNA Biol.* 11 1180–1188. 10.4161/rna.36281 25483034 PMC5155352

[B42] Li Z. Weng H. Su R. Weng X. Zuo Z. Li C. (2017). FTO plays an oncogenic role in acute myeloid leukemia as a *N*^6^-methyladenosine RNA demethylase. *Cancer Cell* 31 127–141. 10.1016/j.ccell.2016.11.017 28017614 PMC5234852

[B43] Liao S. H. Sun H. B. Xu C. (2018). YTH domain: a family of *N*^6^-methyladenosine (m^6^A) readers. *Genomics Proteomics Bioinformatics* 16 99–107. 10.1016/j.gpb.2018.04.002 29715522 PMC6112328

[B44] Lin S. Choe J. Du P. Triboulet R. Gregory R. I. (2016). The m^6^A methyltransferase METTL3 promotes translation in human cancer cells. *Mol. Cell* 62 335–345. 10.1016/j.molcel.2016.03.021 27117702 PMC4860043

[B45] Liu N. Pan T. (2016). *N*^6^-methyladenosine–encoded epitranscriptomics. *Nat. Struct. Mol. Biol.* 23 98–102. 10.1038/nsmb.3162 26840897

[B46] Luo G. Z. MacQueen A. Zheng G. Duan H. Dore L. C. Lu Z. (2014). Unique features of the m^6^A methylome in *Arabidopsis thaliana*. *Nat. Commun.* 5:5630. 10.1038/ncomms6630 25430002 PMC4248235

[B47] Luo S. Tong L. (2014). Molecular basis for the recognition of methylated adenines in RNA by the eukaryotic YTH domain. *Proc. Natl. Acad. Sci. U.S.A.* 111 13834–13839. 10.1073/pnas.1412742111 25201973 PMC4183320

[B48] Ma S. Gong Q. Bohnert H. J. (2006). Dissecting salt stress pathways. *J. Exp. Bot.* 57 1097–1107. 10.1093/jxb/erj098 16510518

[B49] Martínez-Pérez M. Aparicio F. López-Gresa M. P. Bellés J. M. Sánchez-Navarro J. A. Pallás V. (2017). *Arabidopsis* m^6^A demethylase activity modulates viral infection of a plant virus and the m^6^A abundance in its genomic RNAs. *Proc. Natl. Acad. Sci. U.S.A.* 114 10755–10760. 10.1073/pnas.1703139114 28923956 PMC5635872

[B50] Mauer J. Jaffrey S. R. (2018). FTO, m^6^A_m_, and the hypothesis of reversible epitranscriptomic mRNA modifications. *FEBS Lett.* 592 2012–2022. 10.1002/1873-3468.13092 29754392

[B51] Mauer J. Luo X. Blanjoie A. Jiao X. Grozhik A. V. Patil D. P. (2017). Reversible methylation of m^6^A_m_ in the 5′ cap controls mRNA stability. *Nature* 541 371–375. 10.1038/nature21022 28002401 PMC5513158

[B52] Merret R. Nagarajan V. K. Carpentier M.-C. Park S. Favory J.-J. Descombin J. (2015). Heat-induced ribosome pausing triggers mRNA co-translational decay in *Arabidopsis thaliana*. *Nucleic Acids Res.* 43 4121–4132. 10.1093/nar/gkv234 25845591 PMC4417158

[B53] Meyer K. D. Jaffrey S. R. (2014). The dynamic epitranscriptome: *N*^6^-methyladenosine and gene expression control. *Nat. Rev. Mol. Cell Biol.* 15 313–326. 10.1038/nrm3785 24713629 PMC4393108

[B54] Meyer K. D. Jaffrey S. R. (2017). Rethinking m^6^A readers, writers and erasers. *Annu. Rev. Cell Dev. Biol.* 33 319–342. 10.1146/annurev-cellbio-100616-060758 28759256 PMC5963928

[B55] Meyer K. D. Patil D. P. Zhou J. Zinoviev A. Skabkin M. A. Elemento O. (2015). 5′ UTR m^6^A promotes cap-independent translation. *Cell* 163 999–1010. 10.1016/j.cell.2015.10.012 26593424 PMC4695625

[B56] Mielecki D. Zugaj D. L. Muszewska A. Piwowarski J. Chojnacka A. Mielecki M. (2012). Novel AlkB dioxygenases- alternative models for *in silico* and *in vivo* studies. *PLoS One* 7:e30588. 10.1371/journal.pone.0030588 22291995 PMC3265494

[B57] Müller T. A. Struble S. L. Meek K. Hausinger R. P. (2018). Characterization of human AlkB homolog 1 produced in mammalian cells and demonstration of mitochondrial dysfunction in ALKBH1-deficient cells. *Biochem. Biophys. Res. Commun.* 495 98–103. 10.1016/j.bbrc.2017.10.158 29097205 PMC5736403

[B58] Nelson D. C. Flematti G. R. Riseborough J. A. Ghisalberti E. L. Dixon K. W. Smith S. M. (2010). Karrikins enhance light responses during germination and seedling development in *Arabidopsis thaliana*. *Proc. Natl. Acad. Sci. U.S.A.* 107 7095–7100. 10.1073/pnas.0911635107 20351290 PMC2872431

[B59] Nicastro G. Taylor I. A. Ramos A. (2015). KH-RNA interactions: back in the groove. *Curr. Opin. Struct. Biol.* 30 63–70. 10.1016/j.sbi.2015.01.002 25625331

[B60] Niessen M. Schneiter R. Nothiger R. (2001). Molecular identification of virilizer, a gene required for the expression of the sex-determining gene sex-lethal in *Drosophila melanogaster*. *Genetics* 157 679–688. 11156988 10.1093/genetics/157.2.679 PMC1461513

[B61] Oerum S. Dégut C. Barraud P. Tisné C. (2017). m^1^A post-transcriptional modification in tRNAs. *Biomolecules* 7:E20. 10.3390/biom7010020 28230814 PMC5372732

[B62] Ougland R. Lando D. Jonson I. Dahl J. A. Moen M. N. Nordstrand L. M. (2012). ALKBH1 is a histone H2A dioxygenase involved in neural differentiation. *Stem Cells* 30 2672–2682. 10.1002/stem.1228 22961808 PMC3546389

[B63] Ougland R. Rognes T. Klungland A. Larsen E. (2015). Non-homologous functions of AlkB homologs. *J. Mol. Cell Biol.* 7 494–504. 10.1093/jmcb/mjv029 26003568

[B64] Pan Y. Ma P. Liu Y. Li W. Shu Y. (2018). Multiple functions of m^6^A RNA methylation in cancer. *J. Hematol. Oncol.* 11:48. 10.1186/s13045-018-0590-8 29587823 PMC5870302

[B65] Park C. H. Hong K. (2017). Epitranscriptome: m^6^A and its function in stem cell biology. *Genes Genomics* 39 371–378. 10.1007/s13258-016-0507-2

[B66] Pastore C. Topalidou I. Forouhar F. Yan A. C. Levy M. Hunt J. F. (2012). Crystal structure and RNA biding properties of the RNA recognition motif (RRM) and AlkB domains in human AlkB homolog 8 (ABH8), an enzyme catalyzing tRNA hypermodification. *J. Biol. Chem.* 287 2130–2143. 10.1074/jbc.M111.286187 22065580 PMC3265892

[B67] Ping X. L. Sun B. F. Wang L. Xiao W. Yang X. Wang W. J. (2014). Mammalian WTAP is a regulatory subunit of the RNA *N*^6^-methyladenosine methyltransferase. *Cell Res.* 24 177–189. 10.1038/cr.2014.3 24407421 PMC3915904

[B68] Růžička K. Zhang M. Campilho A. Bodi Z. Kashif M. Saleh M. (2017). Identification of factors required for m^6^A mRNA methylation in *Arabidopsis* reveals a role for the conserved E3 ubiquitin ligase HAKAI. *New Phytol.* 215 157–172. 10.1111/nph.14586 28503769 PMC5488176

[B69] Safra M. Saschen A. Nir R. Winkler R. Nachshon A. Bar-Yaacov D. (2017). The m^1^A landscape on cytosolic and mitochondrial mRNA at single-base resolution. *Nature* 551 251–255. 10.1038/nature24456 29072297

[B70] Saletore Y. Meyer K. Korlach J. Vilfan I. D. Jaffrey S. Mason C. E. (2012). The birth of the Epitranscriptome: deciphering the function of RNA modifications. *Genome Biol.* 13:175. 10.1186/gb-2012-13-10-175 23113984 PMC3491402

[B71] Schaefer M. Pollex T. Hanna K. Lyko F. (2009). RNA cytosine methylation analysis by bisulfite sequencing. *Nucleic Acids Res.* 37:e12. 10.1093/nar/gkn954 19059995 PMC2632927

[B72] Schwartz S. Mumbach M. R. Jovanovic M. Wang T. Maciag K. Bushkin G. G. (2014). Perturbation of m^6^A writers reveals two distinct classes of mRNA methylation at internal and 50 sites. *Cell Rep.* 8 284–296. 10.1016/j.celrep.2014.05.048 24981863 PMC4142486

[B73] Scutenaire J. Deragon J. M. Jean V. Benhamed M. Raynaud C. Favory J. J. (2018). The YTH domain protein ECT2 is an m^6^A reader required for normal trichome branching in Arabidopsis. *Plant Cell* 30 986–1005. 10.1105/tpc.17.00854 29618631 PMC6002185

[B74] Shah J. C. Clancy M. J. (1992). IME4, a gene that mediates MAT and nutritional control of meiosis in *Saccharomyces cerevisiae*. *Mol. Cell. Biol.* 12 1078–1086. 10.1128/mcb.12.3.1078 1545790 PMC369539

[B75] Sharma S. Lafontaine D. L. J. (2015). View from a bridge’: a new prospective on eukaryotic rRNA base modification *Trends Biochem. Sci.* 40 560–575. 10.1016/j.tibs.2015.07.008 26410597

[B76] Shen F. Huang W. Huang J. T. Xiong J. Yang Y. Wu K. (2014). Decreased *N*^6^-methyladenosine in peripheral blood RNA from diabetic patients is associated with FTO expression rather than ALK BH5. *J. Clin. Endocrinol. Metab.* 100 148–154. 10.1210/jc.2014-1893 25303482 PMC5399497

[B77] Shen L. Liang Z. Gu X. Chen Y. Teo Z. W. N. Hou X. (2016). *N*^6^-methyladenosine RNA modification regulates shoot stem cell fate in *Arabidopsis*. *Dev. Cell* 38 186–200. 10.1016/j.devcel.2016.06.008 27396363 PMC6364302

[B78] Shi H. Xiao W. Lu Z. Zhao B. S. Ma H. Hsu P. J. (2017). YTHDF3 facilitates translation and decay of *N*^6^-methyladenosine-modified RNA. *Cell Res.* 27 315–328. 10.1038/cr.2017.15 28106072 PMC5339834

[B79] Sledz P. Jinek M. (2016). Structural insights into the molecular mechanism of the m^6^A writer complex. *eLife* 5:e18434. 10.7554/eLife.18434 27627798 PMC5023411

[B80] Sloan K. E. Warda A. S. Sharma S. Entian K. D. Lafontaine D. L. J. Bohnsack M. T. (2017). Tuning the ribosome: the influence of rRNA modification on eukaryotic ribosome biogenesis and function. *RNA Biol.* 14 1138–1152. 10.1080/15476286.2016.1259781 27911188 PMC5699541

[B81] Song J. Zhai J. Bian E. Song Y. Yu J. Ma C. (2018). Transcriptome-wide annotation of m^5^C RNA modifications using machine learning. *Front. Plant Sci.* 9:519. 10.3389/fpls.2018.00519 29720995 PMC5915569

[B82] Squires J. E. Patel H. R. Nousch M. Sibbritt T. Humphreys D. T. Parker B. J. (2012). Widespread occurrence of 5-methylcytosine in human coding and non-coding RNA. *Nucleic Acids Res.* 40 5023–5033. 10.1093/nar/gks144 22344696 PMC3367185

[B83] Tang C. Klukovich R. Peng H. Wang Z. Yu T. Zhang Y. (2018). ALKBH5-dependent m^6^A demethylation controls splicing and stability of long 3′-UTR mRNAs in male germ cells. *Proc. Natl. Acad. Sci. U.S.A.* 115 E325–E333. 10.1073/pnas.1717794115 29279410 PMC5777073

[B84] Ueda Y. Ooshio I. Fusamae Y. Kitae K. Kawaguchi M. Jingushi K. (2017). AlkB homolog 3-mediated tRNA demethylation promotes protein synthesis in cancer cells. *Sci. Rep.* 7:42271. 10.1038/srep42271 28205560 PMC5304225

[B85] Väre V. Y. Eruysal E. R. Narendran A. Sarachan K. L. Agris P. F. (2017). Chemical and conformational diversity of modified nucleosides affects tRNA structure and function. *Biomolecules* 7:E29. 10.3390/biom7010029 28300792 PMC5372741

[B86] Wang C. Y. Shie S. S. Wen M. S. Hung K. C. Hsieh I. C. Yeh T. S. (2015a). Loss of FTO in adipose tissue decreases ANGPTL4 translation and alters triglyceride metabolism. *Sci. Signal.* 8:ra127. 10.1126/scisignal.aab3357 26671148

[B87] Wang X. Feng J. Xue Y. Guan Z. Zhang D. Liu Z. (2016). Structural basis of *N*^6^-adenosine methylation by the METTL3-METTL14 complex. *Nature* 534 575–578. 10.1038/nature18298 27281194

[B88] Wang X. Huang N. Yang M. Wei D. Tai H. Han X. (2017). FTO is required for myogenesis by positively regulating mTOR-PGC-1α pathway-mediated mitochondria biogenesis. *Cell Death Dis.* 8:e2702. 10.1038/cddis.2017.122 28333151 PMC5386528

[B89] Wang X. Lu Z. Gomez A. Hon G. C. Yue Y. Han D. (2014). *N*^6^-methyladenosine-dependent regulation of messenger RNA stability. *Nature* 505 117–120. 10.1038/nature12730 24284625 PMC3877715

[B90] Wang X. Zhao B. S. Roundtree I. A. Lu Z. Han D. Ma H. (2015b). *N*^6^-methyladenosine modulates messenger RNA translation efficiency. *Cell* 161 1388–1399. 10.1016/j.cell.2015.05.014 26046440 PMC4825696

[B91] Wei L.-H. Song P. Wang Y. Lu Z. Tang Q. Yu Q. (2018). The m^6^A reader ECT2 controls trichome morphology by affecting mRNA stability in Arabidopsis. *Plant Cell* 30 968–985. 10.1105/tpc.17.00934 29716990 PMC6002187

[B92] Westbye M. P. Feyzi E. Aas P. A. Vågbø C. B. Talstad V. A. Kavli B. (2008). Human AlkB homolog 1 is a mitochondrial protein that demethylates 3-methylcytosine in DNA and RNA. *J. Biol. Chem.* 283 25046–25056. 10.1074/jbc.M803776200 18603530 PMC3259822

[B93] Widagdo J. Anggono V. (2018). The m^6^A-epitranscriptomic signature in neurobiology: from neurodevelopment to brain plasticity. *J. Neurochem.* 147 137–152. 10.1111/jnc.14481 29873074

[B94] Wu B. Li L. Huang Y. Ma J. Min J. (2017). Readers, writers and erasers of *N*^6^-methylated adenosine modification. *Curr. Opin. Struct. Biol.* 47 67–76. 10.1016/j.sbi.2017.05.011 28624569

[B95] Wu T. P. Wang T. Seetin M. G. Lai Y. Zhu S. Lin K. (2016). DNA methylation on *N*^6^-adenine in mammalian embryonic stem cells. *Nature* 532 329–333. 10.1038/nature17640 27027282 PMC4977844

[B96] Xiang Y. Laurent B. Hsu C. H. Nachtergaele S. Lu Z. Sheng W. (2017). RNA m^6^A methylation regulates the ultraviolet-induced DNA damage response. *Nature* 543 573–576. 10.1038/nature21671 28297716 PMC5490984

[B97] Xiao W. Adhikari S. Dahal U. Chen Y. S. Hao Y. J. Sun B. F. (2016). Nuclear m^6^A reader YTHDC1 regulates mRNA splicing. *Mol. Cell* 61 507–519. 10.1016/j.molcel.2016.01.012 26876937

[B98] Xu C. Wang X. Liu K. Roundtree I. A. Tempel W. Li Y. (2014). Structural basis for selective binding of m^6^A RNA by the YTHDC1 YTH domain. *Nat. Chem. Biol.* 10 927–929. 10.1038/nchembio.1654 25242552

[B99] Yue Y. Liu J. Cui X. Cao J. Luo G. Zhang Z. (2018). VIRMA mediates preferential m^6^A mRNA methylation in 3′UTR and near stop codon and associates with alternative polyadenylation. *Cell Discov.* 4:10. 10.1038/s41421-018-0019-0 29507755 PMC5826926

[B100] Yue Y. Liu J. He C. (2015). RNA *N*^6^-methyladenosine methylation in post-transcriptional gene expression regulation. *Genes Dev.* 29 1343–1355. 10.1101/gad.262766.115 26159994 PMC4511210

[B101] Zhang C. Samanta D. Lu H. Bullen J. W. Zhang H. Chen I. (2016). Hypoxia induces the breast cancer stem cell phenotype by HIF-dependent and ALKBH5-mediated m^6^A-demethylation of NANOG mRNA. *Proc. Natl. Acad. Sci. U.S.A.* 113 E2047–E2056. 10.1073/pnas.1602883113 27001847 PMC4833258

[B102] Zhang S. Zhao B. S. Zhou A. Lin K. Zheng S. Lu Z. (2017). m^6^A demethylase ALKBH5 maintains tumorigenicity of glioblastoma stem-like cells by sustaining FOXM1 expression and cell proliferation program. *Cancer Cell* 31 591–606. 10.1016/j.ccell.2017.02.013 28344040 PMC5427719

[B103] Zhao X. Yang Y. Sun B. F. Shi Y. Yang X. Xiao W. (2014). FTO-dependent demethylation of *N*^6^-methyladenosine regulates mRNA splicing and is required for adipogenesis. *Cell Res.* 24 1403–1419. 10.1038/cr.2014.151 25412662 PMC4260349

[B104] Zheng G. Dahl J. A. Niu Y. Fedorcsak P. Huang C. M. Li C. (2013). ALKBH5 is a mammalian RNA demethylase that impacts RNA metabolism and mouse fertility. *Mol. Cell* 49 18–29. 10.1016/j.molcel.2012.10.015 23177736 PMC3646334

[B105] Zhong S. Li H. Bodi Z. Button J. Vespa L. Herzog M. (2008). MTA is an *Arabidopsis* messenger RNA adenosine methylase and interacts with a homolog of a sex-specific splicing factor. *Plant Cell* 20 1278–1288. 10.1105/tpc.108.058883 18505803 PMC2438467

[B106] Zhou J. Wan J. Gao X. Zhang X. Jaffrey S. R. Qian S. B. (2015). Dynamic m^6^A mRNA methylation directs translational control of heat shock response. *Nature* 526 591–594. 10.1038/nature15377 26458103 PMC4851248

